# Propranolol-Loaded Limonene-Based Microemulsion Thermo-Responsive Mucoadhesive Nasal Nanogel: Design, In Vitro Assessment, Ex Vivo Permeation, and Brain Biodistribution

**DOI:** 10.3390/gels9060491

**Published:** 2023-06-15

**Authors:** Kawthar K. Abla, Souraya Domiati, Rania El Majzoub, Mohammed M. Mehanna

**Affiliations:** 1 Pharmaceutical Nanotechnology Research Lab, Faculty of Pharmacy, Beirut Arab University, Beirut P.O. Box 11-5020, Lebanon; k.abla@bau.edu.lb; 2 Department of Pharmacology and Therapeutics, Faculty of Pharmacy, Beirut Arab University, Beirut P.O. Box 11-5020, Lebanon; t.domyati@bau.edu.lb; 3 Department of Biomedical Sciences, Faculty of Pharmacy, Lebanese International University, Beirut P.O. Box 11-5020, Lebanon; rania.elmajzoub@liu.edu.lb; 4 Department of Industrial Pharmacy, Faculty of Pharmacy, Alexandria University, Alexandria 21521, Egypt

**Keywords:** brain distribution, BBB, limonene, microemulsion, thermo-sensitive, mucoadhesion, nanogel, nanocarriers, propranolol

## Abstract

Propranolol is the first-line drug for managing migraine attacks. D-limonene is a citrus oil known for its neuroprotective mechanism. Thus, the current work aims to design a thermo-responsive intranasal limonene-based microemulsion mucoadhesive nanogel to improve propranolol efficacy. Microemulsion was fabricated using limonene and Gelucire^®^ as the oily phase, Labrasol^®^, Labrafil^®^, and deionized water as the aqueous phase, and was characterized regarding its physicochemical features. The microemulsion was loaded in thermo-responsive nanogel and evaluated regarding its physical and chemical properties, in vitro release, and ex vivo permeability through sheep nasal tissues. Its safety profile was assessed via histopathological examination, and its capability to deliver propranolol effectively to rats’ brains was examined using brain biodistribution analysis. Limonene-based microemulsion was of 133.7 ± 0.513 nm diametric size with unimodal size distribution and spheroidal shape. The nanogel showed ideal characteristics with good mucoadhesive properties and in vitro controlled release with 1.43-fold enhancement in ex vivo nasal permeability compared with the control gel. Furthermore, it displayed a safe profile as elucidated by the nasal histopathological features. The nanogel was able to improve propranolol brain availability with C_max_ 970.3 ± 43.94 ng/g significantly higher than the control group (277.7 ± 29.71 ng/g) and with 382.4 % relative central availability, which confirms its potential for migraine management.

## 1. Introduction

According to the World Health Organization (WHO), neurological diseases are the most hazardous to public health worldwide [1]. Global Burden of Disease (GBD) stated that neurological disorders are the second leading cause of death after cardiac diseases and the main cause of infirmity, affecting approximately 276 million people [2]. Migraine is one of the most common central nervous system (CNS) diseases, characterized by severe pain or a pulsing sensation in one side of the head. It is sometimes accompanied by photophobia, hyperacusis, neurological visual symptoms such as aura, nausea, and vomiting, and the symptoms may last for hours to days. Rather than a vascular headache, migraine was classified recently as a variable, complex disorder of central nervous system function, involving simultaneous alteration in the function of multiple CNS and peripheral nervous components [3].

Efficacious management of migraine requires the elimination of exacerbating factors including stress and inadequate sleep, in addition to acute treatment and preventive approaches. Acute treatment includes triptans, non-steroidal anti-inflammatory drugs, and anti-emetics that are used to reduce the symptoms of migraine attacks [3]. Beta-blockers continue to be a standard therapy for chronic migraine prevention along with acute treatment. Beta-blockers can restrict blood flow to the brain by reducing blood vessel dilation, and they inhibit the nervous system’s electrical activity by making the nervous system less excitable. In addition, they can stabilize serotonin levels in the brain, fluctuations of which are associated with migraine [4]. Among the studied beta-blockers, propranolol (PRO) is considered the most effective in preventing migraine attacks and has been given the highest rating by the Canadian Headache Society (CHS), American Headache Society/American Association of Neurology (AAN/AHS), and the European Federation of Neurological Societies (EFNS) guidelines [5]. Commercially, propranolol is available as tablets and intravenous injections. Low oral bioavailability, hepatic first-pass metabolism, and frequent dosing are the foremost barriers to conventional delivery of PRO [6]. It was reported that PRO has a systemic bioavailability between 15 and 23% and time to peak plasma level T_max_ range between 1 and 2 h after oral administration, which can vary up to 7-fold depending on patient hepatic metabolic activity [7]. Furthermore, biological barriers limit the benefits of propranolol for managing migraine disorder, specifically the blood–brain barrier (BBB), since it hinders PRO passage from the blood to the brain and thus reduces its therapeutic efficacy. Therefore, to overcome the challenges of drug delivery to the brain and design a potent therapeutic strategy, thermo-responsive nanogel has been developed for intranasal delivery of drugs facing the BBB [8].

Temperature- or thermo-responsive nanogel is a modern dosage form that displays sol-gel transition after administration into the body cavity in response to physiological environmental changes. Different smart thermosensitive polymers are utilized for nasal nanogel fabrication. Among them, poloxamer 407 copolymer, known commercially as Pluronic^®^F 127 (P-407), is widely used owing to its ability to transform from solution into a semi-solid gel (sol–gel) at lower concentrations near body temperature [9]. Furthermore, P-407 is well known for its safe nature, local inertia, and prolongation of drug residence time, that enables it to be a suitable candidate for intranasal, ocular, and inner ear drug delivery [10,11,12]. Usually, P-407 is used in combination with other polymers such as chitosan since it easily loses its structural integrity when exposed to aqueous solutions [13]. Chitosan occurs naturally as a cationic linear polysaccharide with a unique ability to form polyelectrolyte complexes due to the presence of an amine group, making it an appropriate component for nanogel formation. Positively charged chitosan can interact with the negatively charged nasal mucosa to form a viscous gel with ideal mucoadhesive properties that ameliorate the drug’s nasal retention. Additionally, it can open the epithelial cell lining tight junction, thus promoting paracellular drug transport [14].

Despite the strong points of the nose-to-brain drug delivery system, the smaller surface area of the nasal cavity compared to the oral one limits the molecules’ absorption. Furthermore, the thin lining of mucous surrounding the nasal cavity has inherent lipophilicity, allowing only the transport of small lipophilic species [15]. In this context, a suitable design of drug nanocarrier loaded within thermo-responsive nanogel has become essential to attain the desired therapeutic effect in the brain. Microemulsions (MEs) have drawn unique attention due to their nanometric globule size, good solubilizing properties, and their potential to incorporate both lipophilic and hydrophilic drug molecules, in addition to their capability to boost drug permeation via biological membranes and prevent degradation by enzymes [16]. Recently, the concept of “essential oils”-based MEs has gained importance amongst researchers worldwide. These oils can enrich drug delivery systems with their intrinsic properties [17]. Limonene oil, for example, is a member of the terpene family that shows various biological properties such as anti-inflammatory, anti-cancer, antioxidant, and gastroprotective characteristics. There are many studies further demonstrating that limonene can be used as an adjuvant to modern therapeutic drugs to slow the course of neurological disease [18]. It was demonstrated that limonene limited the spread of nerve injury that amplified mechanical hyperalgesia in mice that received glycoprotein [19]. These data illustrate the ability of limonene to control migraine attack by reducing hyperalgesia.

In view of this, the current work aims to design a microemulsion-based thermo-responsive mucoadhesive nanogel, taking advantage of limonene oil as a component of the microemulsion oily phase, and using poloxamer-407 and chitosan as thermosensitive and mucoadhesive polymers, respectively, to achieve maximum propranolol nasal permeation and brain biodistribution, aiming to influence chronic migraine. The optimized in situ nanogel was fabricated and assessed regarding its gelation temperature and time, pH, rheological properties, mucoadhesive strength, in vitro release, and ex vivo permeation through sheep nasal tissue using diffusion cells. Additionally, the in situ nanogel safety profile was addressed through histopathological examination. Finally, a study of brain availability was performed using Wistar rat model to evaluate the potency of such formulation to deliver propranolol through the nasal mucosal membrane and across the blood–brain barrier.

## 2. Results and Discussion

### 2.1. Fabrication and Characterization of Propranolol-Loaded Limonene-Based Microemulsion

Recently, the applications of nanotechnology in drug delivery systems have been expanded with revolutionary effects in the medical and pharmaceutical fields. A breakthrough in nanotechnology has been made possible by lipid-based nanocarriers due to their impressive properties, including biocompatibility, low toxicity, and high versatility [20,21,22]. Furthermore, lipid-based nanocarriers can be used to empower the therapeutic potential of active pharmaceutical ingredients that have biopharmaceutical limitations exemplified by poor aqueous solubility, first-pass metabolism, or low stability. Lipid-based nanocarriers include different types of vehicles among which microemulsion is of great interest since it is based on nano-droplet adsorption that can form a reservoir and limit drug drainage [23]. ME is a thermodynamically stable system, composed of oil, water, surfactant, and most of the time co-surfactant that decreases its surface tension. In the present work, limonene oil was incorporated with Gelucire^®^ as the oily phase, along with Labrasol^®^ and Labrafil^®^ as surfactant and co-surfactant, respectively, to design a nanosized microemulsion by spontaneous emulsification technique where propranolol was dissolved in the aqueous phase due to its hydrophilic nature. Limonene, a member of the terpene family, is a natural and safe oil that shows various biological properties including its ability to control migraine attacks by reducing hyperalgesia [18]. Moreover, essential oils, including limonene, are considered as penetration enhancers that boost drug permeation through biological barriers [24]; thus, limonene oil was selected as a lipid phase component. The prepared PRO-loaded limonene-based ME system was characterized based on its diametric size, polydispersity index (PDI), surface charge, entrapment efficiency, drug loading, surface morphology, and the characteristics of propranolol within the system.

**Figure 1:**
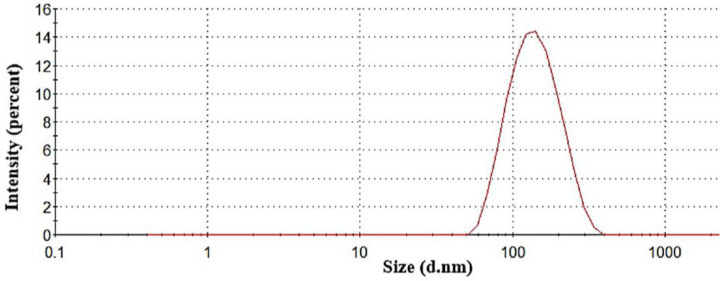
Particle size distribution of propranolol-loaded limonene-based microemulsion.

As illustrated in Figure 1, PRO-loaded limonene-based ME showed a nanometric size (133.7 ± 0.513 nm) and small PDI value (0.112 ± 0.009) due to the inclusion of high Labrasol^®^ and Labrafil^®^ concentrations that raised the entropy of the microemulsion system and decreased the oil/water interfacial tension, which in turn reduced the system’s free energy. The globule size and PDI are important factors for pharmaceutical nanocarriers to bypass biological barriers. Nanosized globules exhibit, in general, a large interfacial surface area that boosts drug permeation through blood–brain barrier [25]. Furthermore, when the nanocarriers’ size is within the range of the gaps of the capillary endothelium present in the basement membrane, the nanocarriers can efficiently pass through the biological barrier [26]. The unimodal particle size distribution of the lipid-based nanovehicles can be detected by PDI, in which homogeneity reflects complete solubilization and incorporation of the different components including the drug, and hence, maximizes drug loading, minimizes the probability of agglomeration, and ameliorates therapeutic efficacy [27]. Relatedly, research performed by Katare et al. proved that microemulsions were able to significantly advance the delivery of Rivastigmine to the brain compared with the control formulation, which was linked to more efficient paracellular and transcellular transport of the microemulsions across the blood–brain barrier [28].

Zeta potential is an important criterion owing to its ability to reflect the physical stability of PRO-loaded limonene-based ME nano-system, in which surface charge values around ±30 mV can offer a large, packed energy barrier which limits globules’ coalescence [29]. Nevertheless, the nanoparticle charge cannot forecast the actual stability profile since the values are solely experience-based [30]. The prepared system showed a negative surface charge with a value of −7.2 ± 1.04 mV, which is considered relatively low. Labrasol^®^, a non-ionic surfactant, caused a transfer of the share plan located in the electrical double layers, resulting in the nanocarrier’s charge reduction [31]. On the other hand, the emulsifier can form a thick film around the oil nano-globules, forming a physical barrier and thus stabilizing the system via steric hindrance rather than electrostatic repulsion [30]. Lin et al. pointed out that the prepared liposomes were sterically stabilized due to the presence of the polymer chain on the liposomes surface rather than the electrostatic effect [32]. Wulff-Perez et al. also drew similar conclusions and stated that inclusion of Pluronic as a non-ionic surfactant in submicron emulsions produced steric hindrance around the nano-droplets and minimized the metabolic degradation of these systems under duodenal and intravenous conditions [33].

Entrapment efficiency (EE) refers to the percentage of the drug encapsulated within the nanocarrier, whereas drug loading (DL) capacity is the amount of drug loaded per unit weight of the formulation, revealing the weight percentage of the particle due to the encapsulated drug. Both parameters indicate the effectiveness of drug incorporation into the carrier and are important when assessing the physical properties of microemulsion [34]. Moreover, high EE and DL influence greatly the rate of drug release and guarantee maximum efficacy of the encapsulated drug at lower doses compared to their free counterpart administration, thereby reducing the drug’s toxic side effects. The prepared ME system showed high entrapment efficiency (81.9 ± 3.02%) and high drug loading capacity (117 ± 0.74 mg·g^−1^). Such high EE and DL are presumably ascribed to the suitable coordination between the drug and the ME components, confirming that propranolol is well-solubilized and entrapped within the microemulsion system with a minimum chance of undesirable leakage [35].

**Figure 2:**
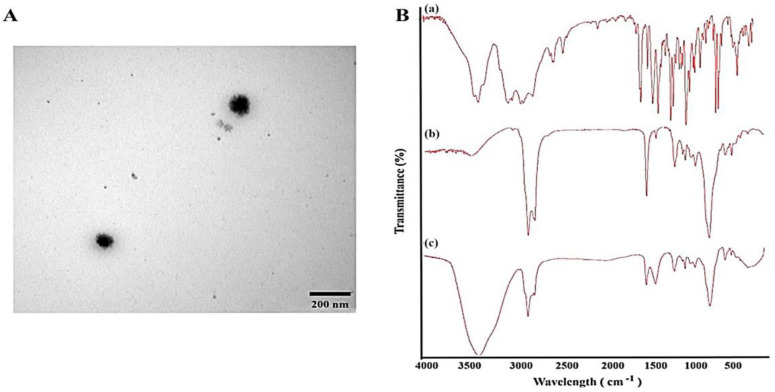
Characteristics of propranolol-loaded limonene-based microemulsion system. (**A**) Transmission electron microscopical image of the optimized propranolol-loaded limonene-based microemulsion system, and (**B**) Fourier transform infrared spectra of (a) propranolol, (b) physical mixture of ME components, and (c) propranolol-loaded limonene-based ME.

Morphological and structural features of PRO-loaded limonene-based ME were examined utilizing TEM, as shown in Figure 2A. The micrograph displayed separate nano-droplets with smooth spherical outlines. Most ME droplets had a diametric size in the range of 150 nm, verifying the data recorded by the Malvern Zetasizer. Likewise, prepared retinoic acid-loaded ME in a previous study displayed separate nano-droplets with smooth outlines [36].

The FTIR spectra of the raw drug, propranolol-loaded limonene-based MEs, and their physical mixture are elucidated in Figure 2B and were utilized to detect chemical compatibility in terms of any possible interaction between excipients and drug. The spectrum of the pure propranolol drug (Figure 2B-a) revealed a characteristic peak corresponding to a secondary amine –N–H stretch at 3202 cm^−1^, C–H stretch at 2950 cm^−1^, and an aryl C=C stretch at 1590 cm^−1^. Moreover, a peak at 1200 cm is attributed to aryl O-CH_2_ asymmetric stretching and another peak at 1010 corresponds to aryl O–CH_2_ symmetric stretching. Finally, a peak was observed at 780 cm^−1^ due to alpha-substituted naphthalene [37]. For PRO-loaded limonene-based MEs and its physical mixture, the FTIR spectra revealed that the characteristic functional groups of PRO were retained, with no new peaks/bands appearance. As a result, the presence of other excipients, such as limonene, Gelucire^®^, Labrasol^®^, and Labrafil^®^, had no effect on the main propranolol peaks. This means there were no chemical changes or interactions between the drug and the excipients that were utilized.

### 2.2. Fabrication and Characterization of Thermo-Responsive Nanogel

Thermo-responsive nanogel responds to temperature change and is converted from sol to gel form. Thus, it represents an attractive approach that reduces the administrated dose outflow through mucociliary clearance, promotes the molecules’ retention in the nasal cavity, controls drug release, and enhances its absorption. It is constituted of thermosensitive polymers that display a sol–gel transformation temperature range between 28 and 36 °C. This is known as the lower critical solution temperature below which hydrogen bonds between the polymer hydrophilic group and water molecules boost polymer chain dissolution, allowing the system to remain in the liquid state. As the temperature increases above this range, the hydrogen bond dissociates, resulting in hydrophobic interaction that favors sol–gel transformation [38]. Poloxamer gelation mechanism is correlated with the intrinsic micellar behavior changes and micelles entanglement at body temperature that shifts the methyl group orientation, resulting in water expulsion from the micelles core. Nevertheless, low viscosity, poor mechanical properties, weak bioadhesion, and rapid erosion limit poloxamer application and require its combination with other polymers such as chitosan [8]. Chitosan is a cationic linear polysaccharide derived from crustacean shells solubilized in acidic media, resulting in the formation of positively charged molecules via amine group protonation within its structure, making it able to form polyelectrolyte complexes [8].

Thermo-responsive nanogel was characterized regarding its gelation temperature, time, strength, surface pH, viscosity, and mucoadhesion properties to ensure its safe and effective profile. The reported temperature in human nasal tissues ranged from 30.2 ± 1.7 °C to 34.4 ± 1.1 °C [39]. The formulation remains in the liquid state at temperatures below 30 °C, causing drainage from the nasal cavity, and the gel forms at temperatures above 34 °C, making it difficult to prepare and administer. In the same manner, short gelling time in the nasal cavity is important to avoid rapid clearance of the liquid formulation and to augment the drug’s residence time in the nasal tissues. Thus, twelve different preparations with different poloxamer and chitosan concentrations were prepared (Table 1), from which the optimized formula (F10) with 26 % *w*/*w* poloxamer and 1% *w*/*w* chitosan was selected for further evaluation, displaying sol–gel phase transition in the temperature range of 30–34 °C with the shortest gelling time. It was clear from the obtained data that a fundamental role is played by chitosan for a rapid and effectual gelling process. This is because there are numerous amino groups (-NH_2_) and hydroxyl groups (-OH) along the chitosan chains that can be used as cross-linkable functional groups to react with poloxamer functional groups to promote in situ hydrogel formation even in the absence of cross linkers [40].

Gel strength is another important factor to be considered when developing a thermo-responsive semi-solid nasal formulation. Since the gel strength is reflected by the time needed by a weight placed on the top of the gel to pass from the gel surface to the bottom of a cylinder [41], gels that take less than 25 s may erode rapidly since they cannot maintain their integrity for sufficient time; on the other hand, gels with strength values greater than 50 s may irritate the mucosal surface due to their stiffness [42]. The optimally prepared gel, F10, revealed an acceptable strength of value 42 s.

The selected PRO in situ nanogel had a pH value of 6.08 ± 0.01. This value indicates that the prepared gel should not cause irritation when applied to the nasal mucosa [43]. Moreover, PRO in situ nanogel displayed a good flow before gelation as shown by its viscosity value, which was in the range of 507 ± 52 cpr at 25 ± 0.5 °C. After gelation, there was a significant increase in the viscosity (2308 ± 125 cpr) at 34 ± 0.5 °C. At low temperatures, the polymeric molecules are hydrated, and there is little interaction between them excluding entanglement. At 34 °C, the polymer chains start to lose their water content, and polymer–polymer interaction starts to take place, resulting in an infinite network structure, which was detected experimentally via the elevation in gel viscosity [44]. Additionally, the drug content of the freshly prepared formulation was found to be about 89 ± 2.3%, confirming the adequacy of the employed preparation technique which led to complete homogeneity and miscibility between the gel components in addition to the negligible loss of propranolol during preparation.

To screen formulations for ex vivo experiments, mucoadhesion is frequently studied in vitro by investigating the possible interactions between the formulation and mucin on a molecular or colloidal scale. The mucoadhesive property of nasal gel refers to the adhesion between the drug carrier and the mucus layer that lines the epithelial cell and/or soft tissue mucosa. Mucoadhesion augments the formulation’s residence time in the nasal cavity [42]. As a result, the mucin particle technique was selected in the current study. The change in the mucin particle charge in the presence of a specific polymeric solution was determined and applied as a reference for the mucoadhesive strength assessment [44]. In the current study, the fabricated mucin suspension revealed a negative zeta potential value of −10.3 mV. This negative value is correlated with mucin carboxyl groups’ ionization, as previously reported. The inclusion of the prepared gel resulted in a significant decrease in the mucin zeta potential value (−3.9 mV), reflecting the interaction and the adhesion of chitosan on the surface of the mucin particles and thus revealing mucoadhesive properties. Similarly, Lin et al. deduced that the change in zeta potential of original mucin particles resulted from the adsorption of the polymer on the surface of the mucin particles, which changed the characteristics of the latter [45].

### 2.3. In-Vitro Release and Ex-Vivo Permeation

**Figure 3:**
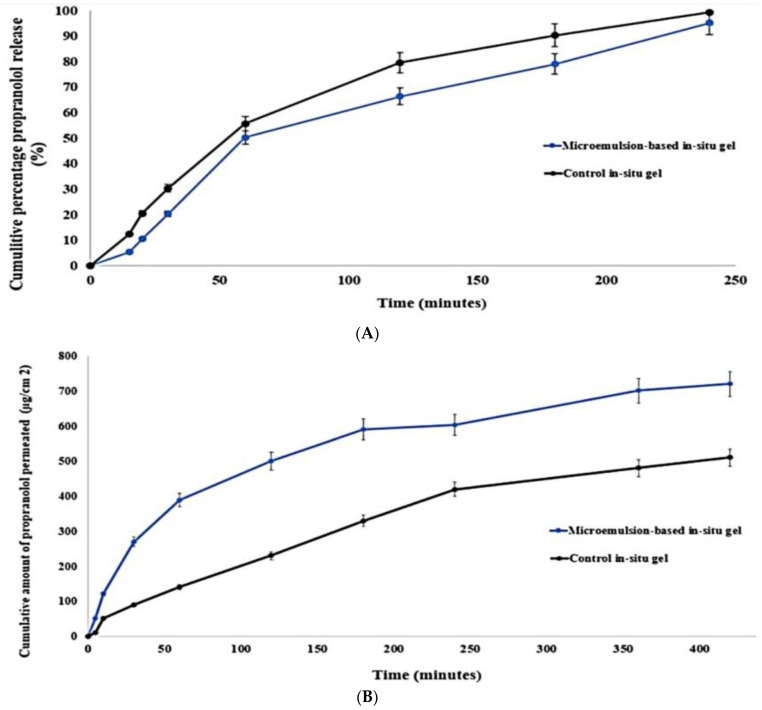
In vitro release pattern (**A**) and ex vivo permeation behavior (**B**) of limonene-based microemulsion thermo-responsive in situ nanogel through a synthetic semi-permeable membrane and sheep nasal tissue, respectively.

Drug absorption and therapeutic response are dependent on the drug release profile from in situ nanogels. Thereby, in situ nasal gel formulations must be meticulously formulated so that the desired drug is released across the nasal mucosa by prolonging the residence time in the nasal cavity. As illustrated in Figure 3A, both the control gel and the PRO-loaded ME in situ nanogel showed controlled release over 4 h. Almost 95% of propranolol was released from the gels within 4 h. The mean release time of PRO-loaded ME in situ nanogel was 1.48 ± 0.1 h, which was longer than that of the control gel (1.21 ± 0.07 h). The release efficiency at 3 h (RE_3_%) of PRO release from the microemulsion-based formulation in situ was 50.42%, significantly lower than that of the control (60.6%). The observed sustained release of both gels contributes to the gel structure that acts as a resistive obstacle to PRO release. Thus, the small number of water channels and high density of the three-dimensional network in the gel structure resulted in prolonged drug release. In addition, at the gelation temperature, the presence of poloxamer in the gel base improved the entanglement, which resulted in high gel strength and led to prolonged drug release. The slower release rate of the prepared PRO-loaded ME-based in situ nanogel in comparison to the control could be linked to the fact that propranolol should first cross the aqueous layer of the microemulsion system then diffuse through the viscous gel structure. A similar observation was reported in the study by Patel et al. in which loteprednol-loaded nanoemulsion in situ nanogel illustrated a prolongation in MRT reflecting a controlled release manner [46]. The slope value ‘n’ of the Korsmeyer–Peppas model confirms the release mechanism. The release component of the in situ gel was found to be 0.79, indicating that the release followed an anomalous model and demonstrating that a combination of diffusion and erosion controlled propranolol release from the polymer matrix.

Fabricating a potential drug carrier requires the use of valid ex vivo models in the early stages of formulation development, with such methods being cheaper, easier, faster, and facing fewer ethical issues than in vivo testing. Because sheep nasal mucosa has similar permeation characteristics to that of humans, it can be used as an ex vivo model for assessing nasal drug permeation [47]. Moreover, it has been documented that sheep nasal mucosa has similar transporter expression, metabolism, and trans-epithelial electrical resistance values to human nasal mucosa [48]. Additionally, it was demonstrated in a study by Gerber et al. that the respiratory epithelial tissue of the sheep shares many common features with the human RPMI 2650 nasal epithelial cell line in terms of drug permeation [49]. Therefore, in the current work, the ex vivo permeation test was conducted on fresh excised sheep nasal mucosa tissue.

In general, there was low correlation between the in vitro release and ex vivo permeation behaviors of both gels, which could be attributed to the differences in nature between the synthetic and the biological membranes. This observation was similar to other previous studies [50,51,52]. Permeation through nasal mucosa was slower for both gels compared with that through cellulose membrane, because of the major barriers exerted by the biological membrane. In contrast to the in vitro release study, propranolol-loaded limonene-based microemulsion in situ nanogel showed enhanced permeation compared with the control gel, as shown in Figure 3B. Based on the cumulative propranolol permeation through the first 4 h (Q_4h_), microemulsion-based in situ nanogel displayed a statistically significant improvement in nasal permeation with respect to the control in situ nanogel. It was observed that more than 600 µg/cm^2^ of propranolol from the ME-based in situ nanogel was permeated through the sheep nasal tissue over 4 h. In contrast, the cumulative amount of propranolol permeated from the control gel was about 400 µg/cm^2^, as illustrated in Figure 3B and Table 2. Furthermore, the steady-state flux (J*_ss_*) and nasal permeability coefficient (P*_eff_*) were around 1.26-fold higher than those of the control in situ nanogel (*p* < 0.05). Additionally, ME-based in-situ nanogel enhanced propranolol permeation by 1.43 times compared with the control gel, indicating that the ME system had a significant major effect on propranolol nasal permeability. This could be explained by the similarity in lipid nature between the nasal endothelium and epithelium layers and the microemulsion components, which facilitated the drug permeation through sheep nasal tissue more than the control gel. Lipid-based nanocarriers, in general, can also ameliorate drug internalization by phospholipids that are predominant in endothelial and epithelium cells, through lipid exchange and fusion processes, thus improving nasal permeation [53]. Thereby, the lipophilic structure of the microemulsion system made it more permeable in the lipophilic layers of the nasal tissue which do not exist in the synthetic membrane. Another factor to be considered is the role of penetration enhancement of ME components. The inclusion of limonene, a terpene penetration enhancer, within the microemulsion system resulted in transient alteration in the intercellular ordered structure of the epithelium lipidic layers, thus ameliorating their fluidity [54]. The effect of permeation enhancers appeared obvious in the investigation of Khan et al. in which the presence of anethole in lipidic vesicles significantly boosted ursolic acid permeation through goat nasal mucosa due to its penetration enhancement characteristics in comparison with the control gel [52]. Labrasol^®^ influences the tight junction permeability which could also contribute to the enhanced nasal permeation of propranolol [55]. Moreover, the nano-sized vehicles expanded the surface area available for propranolol permeation through nasal mucosa, in addition to their ability to transport the drug through the epithelium cells via paracellular or transcellular pathways, particularly endocytosis [56]. These factors played a role in enhancing the drug permeation through biological membranes compared with the synthetic ones.

### 2.4. In-Vivo Evaluation

#### 2.4.1. Histopathological Examination

**Figure 4:**
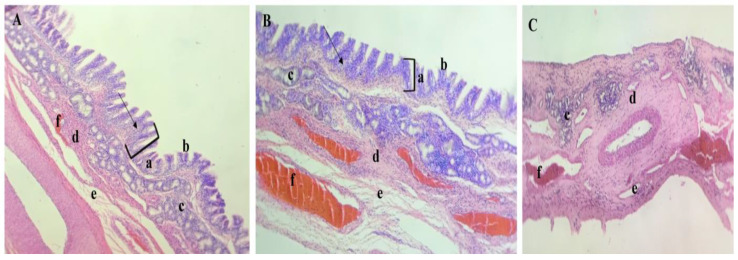
Essential histopathological features of the sheep nasal respiratory tissues. The nasal respiratory tissues of (**A**) the control group and (**B**) the group that received limonene-based in situ nanogel show (a) the classical pseudostratified columnar epithelium layer ciliated with goblet cells (indicated with arrows) and (b) covered from the top with cilia that contain mucosa, opposite to the (**C**) positive treated control group that shows a complete loss of this layer. Different groups show (c) the seromucous glands which secrete their content consisting of mucous and glandular secretions onto the surface the epithelium, to capture bacteria and foreign substances, (d) the connective tissues which surround the mucous glands, and (e) the line of sinusoidal spaces in the endothelium which is normally filled with (f) blood that spreads in between the epithelium.

For accurate evaluation of propranolol-loaded limonene-based microemulsion in situ nanogel safety profile, the histopathological assessment was performed on excised nasal tissue after formulation exposure for six hours. As depicted in Figure 4, the essential features of the sheep nasal respiratory tissues were preserved in the tissue that received the prepared nanogel (Figure 4B), with intact tissue and without any abnormalities, damage, or irritation in the different tissue barriers compared to the control group (Figure 4A). In contrast, the positive control group (Figure 4C) underwent significant changes, specifically epithelium disruption and complete loss in the pseudostratified columnar epithelium layer. This indicates that propranolol-loaded limonene-based microemulsion in situ nanogel can be considered safe with respect to its use for nasal administration.

#### 2.4.2. Brain Uptake Study

**Figure 5:**
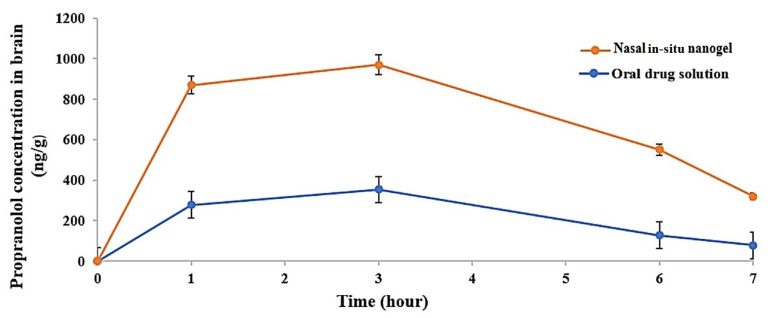
Propranolol behavior in the brain with respect to time after optimized limonene-based microemulsion in situ gel and oral drug solution at a dose of 20 mg/kg (mean values ± SD (*n* = 4)).

To assess the in vivo effectiveness of PRO-loaded limonene-based ME in situ nanogel and the brain biodistribution after intranasal administration, PRO concentration in the brain tissues was determined after oral administration of PRO solution (20 mg/kg) and intranasal administration of PRO-loaded ME in situ nanogel (20 mg/kg). Organ pharmacokinetic parameters were computed by a non-compartmental pharmacokinetic analysis of drug concentrations in the brain up to 7 h post-dosing. Propranolol concentrations, including C_max_, in the brains of the group that received the intranasal gel were significantly higher than those of the oral group at all time points, as illustrated in Figure 5. The AUC_(0–7h)_ of the intranasal treated groups was 4884.15 ± 74.90 ng·h/g, significantly higher than that of the oral treated group which was 1518.35 ± 91.78 ng·h/g, with *p*-value < 0.001 (Table 3). Additionally, t_1/2_ (4.27 ± 1.3) of the intranasal group appeared to be 1.5 times longer than that of the oral group, indicating that the extended retention time in vivo was linked to the adhesive feature of the in situ nanogel. Furthermore, the relative brain availability of the prepared in situ nanogel compared to the oral solution was 382.4%, indicating the ameliorated brain-targeting features of ME-based in situ nanogel. These results cohere with those of the Parashar et al. study in which the nasal administration of lamotrigine-loaded in situ nanogel significantly enhanced the brain availability of the drug compared to its oral suspension [57].

As revealed by the pharmacokinetic study, intranasal ME-based in situ nanogel significantly improved the pharmacokinetic profile of propranolol and augmented its brain availability compared to the oral formulation. This can be explained by the fact that the intranasal route bypasses the hepatic pre-systemic metabolism of propranolol, whereas the oral formulation was unable to protect propranolol from its in vivo metabolic fate, hence it could not reach the brain tissue sufficiently [8].

Once the drug reaches the nasal cavity, it experiences two main fates. It undergoes mucociliary clearance in the vestibular region, and hence the mucoadhesive in situ nanogel is used to reduce this clearance mechanism to reach the nasal posterior region. In the latter region, molecules pass from the nasal cavity into the brain following two main pathways; the first is the systemic pathway where the molecules are absorbed into the systemic circulation through the blood vessels of the respiratory epithelium and cross the blood–brain barrier to reach the brain [58] or through the neuronal pathways where the mechanisms of this pathway can follow many scenarios [57]. It was reported that the nanovehicles can be taken up by trigeminal neurons present in the respiratory epithelium and transported directly into the pons and cerebrum of the brain [59]. In another scenario, nanovehicles can pass through the olfactory region where the drug interacts with the olfactory receptor neurons and moves toward the lamina propria and the brain through the intracellular transport mechanism [60]. In the intracellular transport mechanism, only nanometric vehicles can pass from the olfactory and respiratory epithelium to olfactory sensory neurons and peripheral trigeminal neurons, respectively, through endocytosis. The absorbed molecules can then move through different brain regions through the transcellular pathway which is responsible for the delivery of small lipophilic molecules. This mechanism depends greatly on the size of molecules since it occurs through passive diffusion or endocytosis that requires a nanometric size range [61]. This could be related to the fact that the endothelial cells of the brain are enriched with clathrin vesicles that allow only species below 200 nm to be endocytosed [62]. Thus, owing to many features, limonene-based ME in situ nanogel is considered as an ideal candidate for brain targeting. The microemulsion system offered a sustained drug release profile, as revealed by the ex vivo permeation study, slowing down the absorption rate and increasing the opportunities for nose-to-brain drug transport [63]. It was stated further that the combination of nanovehicle and mucoadhesive in situ nanogel minimizes the chance of mucociliary clearance and increases the formula residence time [64]. In the current study, the inclusion of poloxamer not only promoted the gelation of the formulation at the nasal site, hence elongating the contact time, but also reduced the mucus viscosity and elasticity due to its surfactant properties, leading to lipidic membrane perturbation and improved nasal carrier absorption. In the same manner, positively charged chitosan can interact with the negatively charged nasal mucosa to form a highly viscous gel with ideal bioadhesive characteristics, which in turns ensures the intimate contact between limonene-based microemulsion and nasal tissue, thus augmenting carrier permeation. Furthermore, it is well documented that chitosan can open the tight junctions present within the epithelial cell lining of the nasal cavity, thereby enhancing the drug’s paracellular diffusion [65]. Additionally, the nanometric size of the fabricated microemulsion allows the drug to be transferred from nasal cavity through the olfactory and trigeminal regions to different brain sites by endocytosis [66].

Moreover, the improved brain targeting of propranolol can also be linked to the microemulsion components, namely, lipid and surfactant moieties. In general, transcellular diffusion allows lipophilic nanocarriers to cross the blood–brain barrier, with the rule being that the more lipophilic the molecules, the greater their diffusion into the brain [67]. The inclusion of limonene, a lipophilic terpene with permeation enhancement characteristics, within the microemulsion system resulted in further increasing the fluidity of the nasal and brain lipid bilayers because of the transient alteration in their intercellular ordered structures [54]. On the other side, drugs are delivered into the lumen of cells by transporters which are present in nasal tissues as part of the biological defense barriers, thus restricting drug absorption. The well-known P-glycoprotein (P-gp) is a transporter which exists abundantly in the olfactory submucosa region and on ciliated epithelia, as well as in caveolae of endothelial cells of the blood–brain barrier [68]. It was reported that Labrasol^®^ can inhibit P-gp activity by changing the membrane’s lipid integrity resulting in an interruption in the cell membrane hydrophobic environment, thus restricting P-gp function and improving drug permeation into the brain [69].

### 2.5. Stability Study

The chemical and physical stability of the fabricated in situ nanogel were assessed at two different temperatures. The gel displayed insignificant differences in the parameters assessed. There was no change in its appearance or viscosity before and after gelation of the formulation under the storage conditions for 3 months. Additionally, the gelation temperature and time did not vary significantly and were comparable to the values observed for the fresh formula. Further, there was an insignificant decrease in drug content from 89 ± 2.3% to 88 ± 79% after three months. Hence, it can be deduced that the prepared in situ nanogel displays sufficient stability for three months.

## 3. Conclusions

Propranolol-loaded limonene-based microemulsion (ME) was successfully prepared using limonene and Gelucire^®^ as an oily phase and Labrasol^®^, Labrafil^®^, and deionized water as an aqueous phase. The prepared ME displayed acceptable physicochemical properties including nanometric size (133.7 ± 0.513 nm), narrow PDI (0.112 ± 0.009), and nano-globules with spherical morphological structure. The thermo-responsive microemulsion nanogel was prepared with thermosensitive and mucoadhesive polymers, namely poloxamer and chitosan. The formulated nanogel showed ideal characteristics and was able to ameliorate the ex vivo permeation of propranolol through sheep nasal tissue with a safe profile, as revealed by the histopathological assessment. The brain uptake study revealed that the formulated in situ nanogel can cross the blood–brain barrier, which boosted direct delivery of propranolol to the brain through the nasal route resulting in improved brain availability compared to its oral counterpart. Therefore, it is evident that limonene-based ME in situ nanogel is a conspicuous drug delivery platform for migraine management which can overcome the hepatic first-pass metabolism of propranolol.

## 4. Materials and Methods

### 4.1. Materials

(R)-(+)-Limonene, deacetylated chitin (high molecular weight) chitosan, Poly(ethyleneglycol)-*block*-poly(propylene-glycol)-*block*-poly(ethylenegl col)(Poloxamer 407), and (±)-propranolol hydrochloride were obtained from Sigma Co. (Sigma-Aldrich, Steinheim, Switzerland). Lauroyl polyoxyl-32 glycerides (Gelucire^®^ 44/14), PEG-8 caprylocaproyl macrogol-8 glycerides (Labrasol^®^), and oleoyl polyoxyl-6 glycerides (Labrafil^®^ M 1944 CS) were kindly gifted by Gattefose Co. (Lyon, France). Analytical grade solvents and chemicals were used throughout the investigation.

### 4.2. Methods

#### 4.2.1. Fabrication and Characterization of Propranolol-Loaded Limonene-Based Microemulsion System (PRO-ME)

The microemulsion system was prepared as described in our previous study [70]. Briefly, equal concentrations of Gelucire^®^ and limonene oil were mixed at 47 ± 2 °C for 15 min on a magnetic stirrer (Model KS4L, Falc, Quasi N Co., Ltd., Singapore). Propranolol was dissolved in the aqueous phase consisting of 44.9 % *w*/*w* Labrasol^®^ (surfactant), 11.3% *w*/*w* Labrafil^®^ (co-surfactant), and 0.4 mL deionized water. Under the same stirring conditions, the aqueous phase was then added slowly to the warm oily phase and mixed for another 15 min.

The droplet size, polydispersity index (PDI), and zeta potential of the PRO-ME system were measured utilizing Zetasizer 2000 device (Malvern Instruments, Malvern, UK). The system was diluted 100 times with deionized water before being measured at ambient temperature and at a scattering angle of 90 degree [71].

For determining the entrapment efficiency (EE), PRO-ME was centrifuged for 40 min at 20,000 rpm using a high-speed centrifuge (Sigma- 4L42, Sigma Laboratory Centrifuges, Osteride am Harz, Germany). The supernatant was removed using a micropipette and filtrated before being diluted using methanol to determine free PRO. Entrapment efficiency was computed by the following equation [72]:

(1) $$EE\left(\%\right)=\frac{\left(Total\;amount\;of\;drug\;-\;Free\;drug\;in\;the\;supernatant\right)}{Total\;amount\;of\;drug}\times 100$$

To determine loading capacity, 5 g samples of microemulsion systems were fabricated, with 0.75 g of PRO guaranteed in each formulation. According to the equation below, PRO concentration was computed spectrophotometrically, followed by determining PRO loading within the formulation [73]:

(2) $$Drug\;loading=\frac{Loaded\;drug\left(mg\right)}{Total\;amount\;of\;formulation\left(g\right)}$$

Morphological and structural clarification and the size of propranolol-loaded limonene-based ME were determined via transmission electron microscopy (TEM) (JOE JEM LK 200 electron microscope, Tokyo, Japan). The ME system was diluted with deionized water before a drop of the diluted system was placed on a copper grid. The excess of the sample was removed using a filter paper where the tested sample was then stained with uranyl acetate [74].

Fourier transform infrared spectroscopy (FTIR) was applied to assess the state of propranolol and the possible interaction between PRO and the microemulsion components. FTIR spectrometer was used to record FTIR for PRO, physical mixture, and the prepared PRO-loaded limonene-based MEs at room temperature (Bruker Vector 42, Billerica, MA, USA). Dry potassium bromide was mixed with the samples which were then compressed into a disc and scanned over a range of 4000 cm^−1^ [75].

#### 4.2.2. Fabrication of Thermo-Responsive Nanogel

The prepared propranolol-loaded limonene-based microemulsion system was converted into nanogel using polymeric dispersion. Different chitosan weights were allowed to swell in 1% *v*/*v* acetic acid where the optimized fabricated PRO-ME system was added slowly under a constant stirring rate on a hot magnetic stirrer until a homogeneous mixture was obtained [76]. The preparation was cooled to 4 °C before the addition of poloxamer-407 powder. The final preparations were stored at low temperature (<3 °C) for 48 h to ensure the complete solubilization of poloxamer-407 [77]. A small amount of triethanolamine (TEA) was added to neutralize the pH of the formulated nanogels. Meanwhile, the control gel was simply prepared by mixing a specific volume of cold chitosan dispersion with poloxamer-407 dispersion containing the equivalent amount of propranolol. As shown in Table 4, twelve formulations were prepared which were composed of PRO-ME with different ratios of chitosan (0.5–2% *w*/*w*) and poloxamer-407 (20–26% *w*/*w*).

#### 4.2.3. Preparation and Characterization of Thermo-Responsive Nanogel

##### Sol–Gel Transition Temperature, Gelation Time, and Gelation Strength

Gelation temperature of the designed-in situ nanogels was determined as conducted in a previous study [78], where 4 mL of the cold preparation was transferred into a glass vial, sealed with parafilm, and immersed completely in a thermostated bath adjusted to a temperature of 5 ± 1 °C. The bath temperature was then raised gradually at a rate of 0.5 °C/min. The samples were examined to determine gelation temperature when the meniscus was static on slanting by 90 °C [78].

The gelation time was calculated by immersing the glass vial containing 10 mL of the cold prepared samples and a magnetic bar into a water bath maintained at 34 ± 1 °C under constant stirring (30 rpm). Gelation time was recorded as the time at which the magnetic bar stopped [42]. The optimal formulation was selected based on the results of the previous experiment’s determinations and was subjected to further evaluation including gelation strength, pH, viscosity, and drug content.

Gelation strength of the selected formulation was evaluated according to the Saudagar et al. [79] technique with a slight modification. A specific weight of the optimized formula was transferred into a graduated cylinder and left until the sample was completely jellified. A weight of 2 g was then placed on the gel surface using a thread. The time taken for the weight to pass from the gel surface to the bottom of the cylinder reflects the gel strength.

##### pH, Viscosity, and Drug Content Estimation

A calibrated digital pH meter (SkY 14500V Martni Instruments Co., Ltd., Beijing, China) was used to measure the pH of the selected in situ nanogel at room temperature (25 ± 0.2 °C). The in situ nanogel’s viscosity was determined using a Brookfield viscometer (DV-K-Pro, Brookfield, Middleborogh, LN, USA). Gel viscosity was determined at 10 rpm before and after gelation [80]. Estimation of the drug content was further performed by diluting 1 mL of fabricated formulation in 100 mL of deionized water followed by spectrophotometric analysis [81].

##### Mucoadhesive Strength

The mucin particle technique was employed to assess the mucoadhesive strength of propranolol-loaded limonene-based ME in situ nanogel. Bovine mucin was dissolved in phosphate buffer solution (pH = 6.8) to prepare mucin solution. Then, 150 mg of the prepared in situ nanogel was added to 10 mL of the prepared mucin solution and mixed well using a vortex mixer. After incubation for 48 h, the zeta potential of the previously prepared suspension was computed and compared with the zeta potential of the raw bovine mucin suspension. The value change in zeta potential was used to determine the mucoadhesive strength of the investigated in situ nanogel [39].

##### In-Vitro Release Study

The prepared ME-based and control gels (5 mL each) were placed into dialysis bags with semi-permeable membranes (pre-soaked overnight in phosphate buffer) and immersed into the vessels of the USP dissolution apparatus (Model DT 720, Erweka Ltd., Pittlerstraße, Germany) containing phosphate buffer solution (pH.6.8). The bags were immersed under conditions of 34 ± 0.5 °C temperature with paddle stirring at 100 rpm. Aliquots were withdrawn at regular time intervals and the sink condition was maintained by adding the same volume of the release medium [82]. The withdrawn aliquots were spectrophotometrically assayed for their propranolol content.

Based on the following equations, percentage dissolution efficiency (DE%) and mean dissolution time (MT) were computed to compare PRO release from the in situ nanogel and the control.

(3) $$\mathrm{DE}\%=\frac{\int_{t_{1}}^{t_{2}}y\times dt\times 100}{y_{100}\times (t_{2}-t_{1})}$$

(4) $$\mathrm{MDT}=\frac{\sum_{i}^{n}t_{mid}△C}{\sum_{i}^{n}△C}$$

DE is calculated by measuring the area under the release curve between two different times which are expressed as *t*_1_ and *t*_2_. *y*_100_ represents the maximum percentage of drug release between these two points. *y* is computed as the percentage of the drug released at a specific time (*t*), with *t*_1_ = 0 and *t*_2_ = 6 h where 70 to 90% of propranolol was released.

MDT is used in assaying dissolution profiles, in which *n* is the number of release sample times, *i* is the sample number, *t_mid_* is the mid-point time computed by (*t_i_* + *t*_*i*−1_)/2, and △*C* is the additional concentration of the released drug between *i* and *i* − 1.

The release exponent (*n*) value of the Korsmeyer–Peppas model was determined to describe the mechanism release of the drug from the polymer-based preparation.

#### 4.2.4. Ex-Vivo Permeation Study

##### Preparation of Sheep Nasal Mucosa

**Figure 6:**
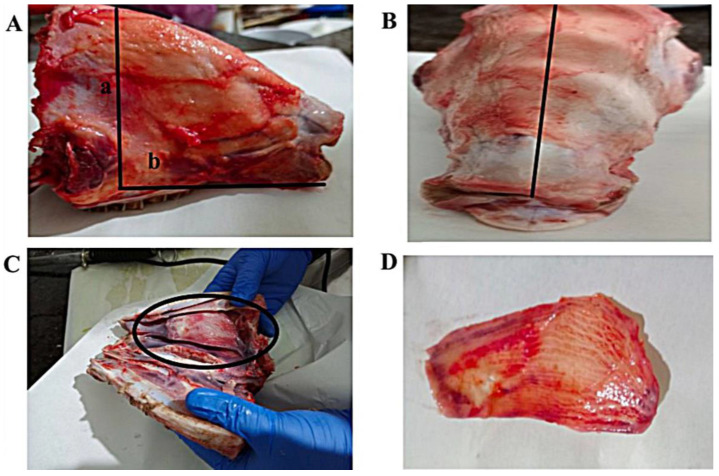
The various steps of the excision of the sheep nasal mucosa. (**A**) An incision anterior to the eyes was adapted to remove the front part of the sheep’s upper jaw as framed by a and b, followed by skin removal via dissection. (**B**) The septum was removed after bisecting the snout along the septal midline. (**C**) Following the separation into two halves, a vertical incision was performed via the snout lateral to detach the conchae, followed by another incision across the conchae to enhance the removal of nasal mucosa tissue. (**D**) Finally, strips were cut from the obtained sheep nasal tissue specimens.

Freshly excised sheep nasal mucosa was collected from a local farm. Figure 6 depicts the steps of the nasal tissue excision technique. As soon as the sheep was slaughtered, the mucosa was carefully removed without the septum and immersed instantly in cold phosphate buffer saline (PBS, pH 6.4) for 30 min to keep the excised nasal tissues viable throughout the experiment. An ex vivo permeation study of PRO-loaded ME in situ nanogel through sheep nasal mucosa was conducted using Franz diffusion cells. Mucosal specimens of 0.10 cm thickness and 3.2 cm^2^ effective areas were utilized for the ex vivo permeation study. In each diffusion cell, the tissue was placed with the mucosa surface facing the donor chamber and the serosal side facing the receptor chamber. The permeability experiment was started by filling the receptor chamber with warm simulated nasal electrolyte solution (SNES) (electrolyte solution consisting of calcium, potassium, and sodium salts of pH 6.5). The Franz cells were placed in a water bath at 34 ± 0.5 °C and at 200 rpm. Before loading the in situ nanogels, the mucosa was allowed to stabilize for 15 min [83].

##### Intranasal Propranolol Delivery

The permeation through the nasal mucosa was studied throughout a period of 6 h after topical exposure of the tested formulation. On the surface of the nasal mucosa, 100 µL of PRO-loaded ME and the control in situ gels (equivalent to 5 mg PRO) were applied. SNES samples were withdrawn at definite time intervals from the receptor chamber and replaced with an equal volume of fresh SNES to maintain sink condition. The samples were quantified spectrophotometrically at 290 nm. The cumulative amount of drug permeated (Q_n_), steady-state flux (J_ss_), trans-nasal permeability coefficient (P_eff_), and enhancement factor (ER) were computed according to the following equations:

(5) $$Qn_{\;}=V_{r}C_{r(n)}+\sum_{x=1}^{x=n}Vs\left(x-1\right)Cr(x-1)$$

(6) Jss=(dQdt)A

(7) $$P_{\mathrm{eff}}=\frac{J_{ss}}{C_{0}}$$

(8) $$\mathrm{ER}=\frac{J_{ss}(Test)}{J_{ss}(Control)}$$

where V_r_: the reservoir compartment volume (mL), V_S_: the sample collected volume (mL) at the nth time point, n: the sampling time point, C_r(n)_: propranolol concentration in the receiver compartment at the nth time point (µg/mL), Q: the amount of transported drug, A: the exposed area of permeation (0.635 cm^2^), and C_0_: initial donor concentration for propranolol.

#### 4.2.5. Assessment of Local Toxicity of the In Situ Nanogel through Histopathological Examination

Histopathological assessment was performed after 6 h of treatment of nasal mucosa with the prepared in situ nanogel, obtained in a similar method to that mentioned in Section 2.4.2. to identify any pathological alteration in tissue and/or cell morphology and organization. The formulations were tested on three nasal tissue samples: untreated mucosa served as the negative control, mucosa treated with 0.1 mL of 38% *v*/*v* nitric acid served as the positive control, and the third mucosa was treated with the prepared thermo-responsive microemulsion-based nanogel. Mucosal tissues first underwent the fixation step using a 10% formaldehyde-based solution, and were then embedded in paraffin, sectioned through the microtome, and placed on microscopical slides. The slides were then dried to remove moisture and de-waxed using xylene. The latter was removed using alcohol and the tissues were hydrated with distilled water. The slides were then stained with hematoxylin and the excess background stain was removed using weak alcohol. Finally, the slides were stained with an alcoholic solution of eosin where a thin layer of polystyrene mountant was applied, followed by a glass coverslip [84].

#### 4.2.6. Brain Uptake Study

##### Experimental Animal

The brain biodistribution study was conducted in albino Wistar rats, which weighed between 130 and 150 g. Rats were obtained from the Faculty of Pharmacy animal house at Beirut Arab University (BAU) in Lebanon. Under standard animal housing conditions, they were kept in polyacrylic cages throughout the experiments. The rats were supplied with water and standard laboratory chow. As authenticated by the Ministry of Public Health, animals were handled according to the Institutional Animal Care and Use Guidelines (IACUG) at BAU, Lebanon. All experiments at Beirut Arab University were approved by the Investigation Review Board (IRB), number 2023-H-0088-P-R-0531.

##### Intranasal and Oral Administration

The pharmacokinetic evaluation was carried out according to the guidelines for the care and use of laboratory animals.

Rats were divided into two groups, each group of 16 rats. In the first group, rats were anesthetized before administration using thiopental through intraperitoneal injection (60 mg/kg i.p.). The selected dose of thiopental has been reported as optimal to invoke the comparable depth of anesthesia assessed by muscle reflex tests. Thiopental was dissolved in 0.9% NaCl and injected at a volume of 1 mL/kg 15 min before the experiment. Animals were fixed and cold formulations (equivalent to 20 mg/kg) were instilled through a polyurethane tube attached to a micropipette. The tubes were inserted about 12 mm deep into one of the nostrils, enabling the delivery of the gels toward the roof of the nasal cavity. Rats remained fixed to ensure complete gelation of the in situ nanogels. The second group received an oral propranolol solution using an oral gavage technique [85].

At a predetermined interval (8 h), rats were sacrificed, and brain samples were harvested and washed with 0.9% sodium chloride solution, dried on tissue paper, and weighed. Then, organs were placed in a glass beaker where phosphate buffer saline of pH 6.8 was added, and the brains were homogenized using a homogenizer. Homogenates were stored at −70 ± 2 °C for further analysis.

##### Extraction of Propranolol from Brain Tissues

Frozen samples were thawed at room temperature (25 ± 2 °C), and 200 µL of samples were added to test tubes followed by the addition of 400 µL of acetonitrile. Tested samples were vortexed for 20 min, then transferred to Eppendorf tubes, and centrifuged at 8500 rpm for 20 min at 5 ± 2 °C to precipitate the proteins. The precipitates were discarded and 100 µL samples of the clear supernatants were transferred to clean dry vials, filtered, sonicated, and assayed with reverse-phase high-performance liquid chromatography [57].

##### HPLC Analysis

An Agilent C18 column (250 × 4.7 mm LD), autosampler, pump, and photodiode array detector were utilized for the quantitative analysis (Waldbronn, Germany). After eluting the isocratic mobile phase consisting of acetonitrile and phosphate buffer of pH 4.5 (35:65) at room temperature (25 ± 0.5 °C), the column effluent was detected at 214 nm wavelength. The retention time of propranolol was found to be 6.5 min. PRO concentration was calculated using a calibration curve plotting the peak area versus concentration [86].

##### Brain Pharmacokinetics 

The pharmacokinetic parameters of propranolol in the brain were determined by applying a non-compartmental approach and Kinetica 4.4.1 SPSS 15 software^®^. The maximum PRO concentration (C_max_, g/mL) and the time at which C_max_ was achieved (T_max_, hours) were calculated using the concentration–time curve. The linear trapezoidal rule was applied to compute the area under the concentration–time curve from zero to the last analyzed point (AUC_0–7_, g·h/mL), and cp/k was added to obtain AUC_0–∞,_ where cp is the last measured concentration and k is the elimination rate constant. The first-order formulation was used to calculate T_1/2_ [87].

#### 4.2.7. Stability Study

The stability of the optimal limonene-based thermo-responsive nanogel was evaluated at temperatures of 25 ± 2 °C (65 ± 5% RH) and at 3 ± 2 °C (50 ± 5% RH) for 12 weeks, according to the ICH guidelines [88]. Samples were stored in glass vials and covered tightly with parafilm. Every four weeks, samples were assessed for appearance, pH, viscosity, gelation temperature and time, and drug content.

#### 4.2.8. Statistical Analysis

Except for in vivo experiment (brain distribution study), which was expressed as mean SEM (standard error of the mean), the results from various in vitro and ex vivo tests were expressed as mean standard deviation (SD) and were analyzed utilizing a one-way analysis of variance (ANOVA) test, followed by a post-hoc Tukey test. When *p*-values were less than 0.05, the results were considered statistically significant. For statistical analysis, the Statistical Package for the Social Sciences (SPSS) version 22 (IBM, New York, NY, USA) was utilized.

## Figures and Tables

**Table 1:** Gelation temperature and gelation time* of the prepared formulations.

Formulation Code	Gelation Temperature °C	Gelation Time (Seconds)
F1	>36	ND **
F2	>36	ND **
F3	>36	ND **
F4	>36	ND **
F5	35.5	55
F6	34.9	49
F7	36.3	52
F8	34.6	48
F9	32.4	25
F10	33.3	35
F11	29.5	15
F12	28	IN *

* Gelation time was measured at 34 ± 0.5 °C; ** IN: instantaneous, and ND: not determined since gelation temperatures were above the accepted range.

**Table 2:** Nasal permeation parameters of propranolol-loaded limonene-based microemulsion thermo-responsive nanogel and the control gel *.

Formula	# Q_4h_ (µg/cm^2^)	# J*_ss_* (µg/cm^2^·min)	# P*_eff_* (cm^2^/min)
ME-based nanogel	603.55 ± 91 ^△^	1.56 ± 0.9 ^△^	0.156 ± 0.03 ^△^
Control gel	420.3 ± 64	1.24 ± 0.4	0.124 ± 0.05

* Values are expressed as mean ± SD; (n = 3). # Q_6h_, J*_ss_*, and P*_eff_* are the cumulative amount of propranolol permeated at 4 h, steady-state flux, and nasal permeability coefficient, respectively. ^△^ the mean difference is significant at *p* < 0.05 compared to the control.

**Table 3:** Pharmacokinetic parameters of propranolol in the brain after oral solution and intranasal microemulsion-based in situ nanogel administration.

Pharmacokinetic Parameters *	Propranolol Solution (Oral Route)	Propranolol Nanogel (Intranasal Route)
C_max_ (ng/g)	277.7 ± 29.71	970.3 ± 43.94
AUC_0–7_ (ng·h/g)	1518.35 ± 91.78	4884.15 ± 74.90
AUC_0–∞_ (ng·h/g)	1829.25 ± 98.01	6995.15 ± 77.39
Ke (h^−1^)	0.369521 ± 0.001	0.25775 ± 0.003
T_max_ (h)	1.89	1.75
t_1/2_ (h)	1.87 ± 0.8	2.69 ± 1.02
Relative availability (%)	-	382.4

* Data are mean values ± SD (*n* = 4).

**Table 4:** Thermo-responsive microemulsion nanogel compositions *.

Formulation	Poloxamer (%*w*/*w*)	Chitosan (%*w*/*w*)
F1	20	0.5
F2	20	1
F3	20	2
F4	22	0.5
F5	22	1
F6	22	2
F7	24	0.5
F8	24	1
F9	24	2
F10	26	0.5
F11	26	1
F12	26	2

* Microemulsion components: 21.9% limonene, 21.9% Gelucire^®^, 44.9% *w*/*w* Labrasol^®^, 11.3% *w*/*w* Labrafil^®^, and 0.4 mL deionized water.

## Data Availability

The data presented in this study are available upon request from the corresponding author.
